# Salicylic acid and Tocopherol improve wheat (*Triticum aestivum* L.) Physio-biochemical and agronomic features grown in deep sowing stress: a way forward towards sustainable production

**DOI:** 10.1186/s12870-024-05180-8

**Published:** 2024-05-30

**Authors:** Saleha Saeed, Sami Ullah, Fazal Amin, Jehad S. Al-Hawadi, Mohammad K. Okla, Ibrahim A. Alaraidh, Hamada AbdElgawad, Ke Liu, Matthew Tom Harrison, Shah Saud, Shah Hassan, Taufiq Nawaz, Mo Zhu, Haitao Liu, Mushtaq Ahmad Khan, Shah Fahad

**Affiliations:** 1 Department of Botany, University of Peshawar, Peshawar, 25120 Pakistan; 2 Faculty of Science, Zarqa University, Zarqa, 13110 Jordan; 3 Botany and Microbiology Department, College of Science, King Saud University, P.O. Box 2455, Riyadh, 11451 Saudi Arabia; 4 Department, College of Science, King Saud University, P.O. Box 2455, Riyadh, 11451 Saudi Arabia; 5 Integrated Molecular Plant Physiology Research, Department of Biology, University of Antwerp, Antwerp, 2020 Belgium; 6 Tasmanian Institute of Agriculture, University of Tasmania, Burnie, TAS 7250 Australia; 7 College of Life Science, Linyi University, Linyi, 276000 Shandong China; 8 Department of Agricultural Extension Education & Communication, The University of Agriculture, Peshawar, 25130 Khyber Pakhtunkhwa Pakistan; 9 Department of Biology and Microbiology, South Dakota State University, Brookings, SD 57007 USA; 10 College of Life Sciences, Henan Normal University, Xinxiang, 453007 P.R. China; 11 Henan International Joint Laboratory of Agricultural Microbial Ecology and Technology, Henan Normal University, Xinxiang, 453007 P.R. China; 12 Xinxiang Key Laboratory of Plant Stress Biology, Xinxiang, 453000 P.R. China; 13 College of Resources and Environment, Henan Agricultural University, Zhengzhou, 450002 PR China; 14 Department of Agriculture, University of Swabi, Khyber Pakhtunkhwa, 23561 Pakistan; 15 Department of Agronomy, Abdul Wali Khan University Mardan, Mardan, 23200 Khyber Pakhtunkhwa Pakistan

**Keywords:** Salicylic acid, Tocopherol, Physio-biochemical, Deep sowing, *Triticum aestivum* L

## Abstract

**Background:**

The rate of germination and other physiological characteristics of seeds that are germinating are impacted by deep sowing. Based on the results of earlier studies, conclusions were drawn that deep sowing altered the physio-biochemical and agronomic characteristics of wheat (*Triticum aestivum* L.).

**Results:**

In this study, seeds of wheat were sown at 2 (control) and 6 cm depth and the impact of exogenously applied salicylic acid and tocopherol (Vitamin-E) on its physio-biochemical and agronomic features was assessed. As a result, seeds grown at 2 cm depth witnessed an increase in mean germination time, germination percentage, germination rate index, germination energy, and seed vigor index. In contrast, 6 cm deep sowing resulted in negatively affecting all the aforementioned agronomic characteristics. In addition, deep planting led to a rise in MDA, glutathione reductase, and antioxidants enzymes including APX, POD, and SOD concentration. Moreover, the concentration of chlorophyll a, b, carotenoids, proline, protein, sugar, hydrogen peroxide, and agronomic attributes was boosted significantly with exogenously applied salicylic acid and tocopherol under deep sowing stress.

**Conclusions:**

The results of the study showed that the depth of seed sowing has an impact on agronomic and physio-biochemical characteristics and that the negative effects of deep sowing stress can be reduced by applying salicylic acid and tocopherol to the leaves.

## Introduction

Wheat is the most widely grown staple crop in the world, used for human nourishment and dietary products. However, traditional cultivation practices and changing climate patterns have caused significant concerns for its growth and sustainable productivity. After rice, it is the second most commonly grown food crop in developed countries. Approximately 40% of the world’s population relies on it as a primary food source, and about 80 million farmers depend on it for their livelihood [1]. In regions where precipitation is scarce, growing wheat in a sustainable manner can be a daunting task, primarily due to the unavailability of water. Suppose the sowing of wheat seeds is postponed because of rainfall, the wheat stands may not mature correctly within the expected timeline, leading to insufficient crop yields. However, according to research, increasing the depth at which the seeds are sown can significantly enhance the growth of wheat seedlings by increasing the amount of water available in the soil of the seed zone. When seeds are sown at greater depths, they are exposed to a more extensive range of soil moisture, which is necessary for better seed germination and establishment. Additionally, deeper seed sowing can protect the seeds from the adverse effects of unfavorable weather conditions, such as drought or frost. Furthermore, deeper seed sowing ensures that the seeds are firmly anchored in the soil, which helps in the efficient absorption of nutrients and water. Therefore, increasing the depth of sowing of wheat seeds can be an effective technique to overcome the challenges of water scarcity and improve crop yields in dry regions [2]. When it comes to planting seeds, it is important to take into account the depth at which they are sown. While deep planting may seem like a good idea to protect seeds from environmental stressors such as drought or high temperatures, it can actually have the opposite effect [3–5]. A phenolic compound called Salicylic acid (SA) regulates plant development and growth as well as how plants react to biotic and abiotic stimuli [6–10]. In order to protect plants from environmental stressors, salicylic acid (SA) regulates crucial physiological processes such as photosynthesis, nitrogen metabolism, proline metabolism, antioxidant defense system, and plant-water interactions [6–9, 11, 12]. Alpha-tocopherol is considered the most effective in neutralizing reactive oxygen species (ROS) and in promoting antioxidant enzymes [13]. The tolerance of plants to stress caused by salt, chilling, UV-B, and pollution is found to be inversely proportional to their tocopherol levels. Alpha-tocopherol, which is a potent antioxidant, supports the integrity of cell membranes, intracellular signaling, and electron transport in the photosystem-II. It also acts as a protectant against photo damage [14]. In Faba bean plants, the foliar application of alpha-tocopherol improved growth metrics, yield constituents, chlorophyll a, b, and carotenoids content [15]. The aim of the current study is to evaluate the agronomic and physio-biochemical responses of economically significant wheat varieties, namely “Pirsabak 15” and “Shankar,” to different concentrations of exogenously applied salicylic acid and alpha-tocopherol. The study also seeks to compare the effectiveness of deep sowing stress management and identify the most effective and robust method. Furthermore, the study aims to determine which variety of wheat is more resilient to deep sowing under exogenously applied salicylic acid and tocopherol.

## Results

### Morphological features

The deep sowing had a negative impact on germination parameters. Germination rate was highest at 2 cm and lowest at 6.0 cm. As the depth of sowing increased, the number of germinated seeds decreased. Similarly, other important agronomic parameters such as GP, CVG, GI, SVI-I, SVI-II, MGT, GE, and GRI also showed a significant decline (Table 1). Moreover, fluctuations were observed in number of leaves of the seedlings in the 8th week of deep sowing. The combined effect of Salicylic acid and alpha-tocopherol alleviated the harmful impacts of deep sowing by enhancing the number of leaves, leaf dry weight, leaf fresh weight, leaf length, % moisture content, leaf area index, leaf area ratio, shoot length, shoot fresh weight, shoot dry weight, root length, and root/shoot ratio. Generally, the seed sowed at depth of 2 cm produced seedling with highest germination, whereas seeds sown at 6 cm depth gave rise to seedlings with lower germination rate. Statistically, a significant decline (*p* ≤ 0.05) was noticed in germination parameter with increasing sowing depth (Table 1). When it comes to maximizing the potential of shoot length, one of the most important factor to consider is the depth of seed sowing. While measuring shoot height of seedlings it was noted that seedlings whose seeds were grown at 2 cm had longer shoot height than those seedlings which were sown at 6 cm depth. It has been found that increasing the depth of sowing caused a significant (*p* < 0.05) reduction in seedling shoot height (Table 2). Statistical analysis revealed that leaf area ratio showed significant (0 < 0.05) changes at varying sowing depths, it showed a marked decrease with increasing the sowing depth (Table 3).

**Table 1:** Impact of salicylic acid and tocopherol foliar spray on (germination index, germination percentage, mean germination time, coefficient velocity of germination, germination energy, germination rate index) of wheat *(Triticum aestivum)* under deep sowing stress

Varieties	Treatments	Germination Percentage (GP)	Germination Index (GI)	Mean Germination Time (MGT)	Coefficient Velocity Of Germination	Germination Energy (GE)	Germination Rate Index (GRI)
**Pirsabak15**	Control	97.3 ± 1.00^a^	827 ± 2.27^a^	13.9 ± 0.08^a^	266 ± 19.9^a^	59.7 ± 1.94^a^	50.5 ± 0.06^a^
	Deep Sowing	83.3 ± 0.00^c^	627 ± 6.13^c^	12.4 ± 0.06^b^	228 ± 6.41^b^	50.3 ± 0.75^c^	44.5 ± 0.82^c^
	Deep Sowing + Salicyclic acid	86.6 ± 0.50^c^	619 ± 5.41^c^	12.3 ± 0.01^b^	213 ± 11.6^c^	48.5 ± 1.24^c^	43.5 ± 1.06^c^
	Deep Sowing + alphaTocopherol	83.3 ± 0.50^a^	618 ± 10.6^c^	12.0 ± 0.00^b^	228 ± 16.9^b^	53.6 ± 1.87^c^	43.5 ± 1.66^c^
	Deep Sowing + Salicyclicacid + Tocopherol	90.0 ± 0.00^b^	675 ± 4.60^c^	13.0 ± 0.08^a^	239 ± 14.9^b^	54.7 ± 1.78^c^	47.0 ± 1.7^b^
**Shankar**	Control	96.6 ± 0.50^a^	721 ± 5.53^c^	13.6 ± 0.06^a^	261 ± 8.08^a^	56.6 ± 0.90^b^	49.7 ± 0.92^a^
	Deep Sowing	86.6 ± 0.50^c^	619 ± 3.28^c^	12.3 ± 0.06^b^	225 ± 11.0^b^	53.5 ± 1.31^b^	41.0 ± 1.32^c^
	Deep Sowing + Salicyclic acid	93.3 ± 0.50^b^	576 ± 6.5^d^	12.2 ± 0.07^b^	218 ± 16.4^b^	49.4 ± 1.87^d^	44.1 ± 1.71^c^
	Deep Sowing + alphaTocopherol	70.0 ± 0.00^d^	636 ± 1.61^c^	12.7 ± 0.07^b^	198 ± 14.7^c^	47.0 ± 1.75^d^	42.0 ± 1.55^c^
	Deep Sowing + Salicyclicacid + Tocopherol	94.6 ± 0.50^b^	646 ± 7.99^c^	12.9 ± 0.00^ab^	231 ± 2.00^b^	54.1 ± 0.33^c^	45.2 ± 0.23^b^

**Table 2:** Impact of salicylic acid and tocopherol foliar spray on (shoot fresh weight, shoot length, shoot dry weight, root length, shoot %moisture content, root/shoot ratio, seed vigor index II, seed vigor index I) of *Triticum aestivum* under deep sowing stress

Varieties	Treatments	Shoot Lenght (cm)	Shoot fresh weight (mg)	Shoot dry weight (mg)	Root Lenght (cm)	%Moisture Content Of Shoot	Root/Shoot Ratio	Seed Vigor Index I (SVI I)	Seed Vigor Index II (SVI II)
**Pirsabak15**	Control	35.6 ± 0.68^a^	999 ± 0.01^a^	460 ± 0.10^a^	13.3 ± 0.29^a^	410 ± 0.76^b^	0.56 ± 0.51^a^	110 ± 1.79^a^	28.3 ± 0.88^a^
	Deep Sowing	23.4 ± 0.80^b^	530 ± 0.04^c^	180 ± 0.07^c^	8.33 ± 1.64^b^	170 ± 0.75^c^	0.33 ± 0.67^c^	84.0 ± 1.15^b^	10.0 ± 1.32^c^
	Deep Sowing + Salicyclic acid	23.3 ± 1.25^b^	450 ± 0.03^d^	100 ± 0.08^d^	8.21 ± 1,29^b^	250 ± 0.86^cd^	0.38 ± 0.48^c^	66.3 ± 0.69^cd^	9.00 ± 0.35^c^
	Deep Sowing + alpha Tocopherol	23.8 ± 2.21^b^	350 ± 0.00^e^	90.0 ± 0.04^ab^	9.33 ± 0.43^bc^	170 ± 0.82^c^	0.59 ± 0.98^a^	68.0 ± 1.60^cd^	6.88 ± 0.61^d^
	DeepSowing + Salicyclicacid + alphaTocopherol	32.6 ± 0.61^de^	730 ± 0.02^b^	390 ± 0.06^b^	10.1 ± 0.36^b^	630 ± 0.63^a^	0.37 ± 0.44^c^	94.1 ± 3.00^ab^	15.3 ± 2.69^bc^
**Shankar**	Control	28.4 ± 1.39^b^	300 ± 0.02^e^	171 ± 0.04^c^	10.9 ± 0.36^b^	268 ± 1.67^c^	0.66 ± 0.29^a^	86.6 ± 0.37^b^	19.9 ± 1.21^b^
	Deep Sowing	19.1 ± 1.20^c^	360 ± 0.06^e^	84.0 ± 0.09^e^	9.16 ± 1.54^bc^	132 ± 0.60^c^	0.32 ± 0.09^c^	60.4 ± 2.07^d^	7.21 ± 1.30^d^
	Deep Sowing + Salicyclic acid	26.1 ± 2.45^b^	440 ± 0.04^d^	101 ± 0.12^d^	10.3 ± 0.84^b^	158 ± 0.67^cd^	0.50 ± 0.16^ab^	60.9 ± 2.11^d^	9.68 ± 1.33^c^
	Deep Sowing + alpha Tocopherol	27.6 ± 2.00^b^	410 ± 0.03^d^	123 ± 0.10^cd^	9.50 ± 0.00^bc^	194 ± 0.59^c^	0.50 ± 0.04^ab^	84.0 ± 1.48^b^	6.69 ± 1.11^d^
	DeepSowing + Salicyclicacid + alphaTocopherol	20.1 ± 0.46^c^	510 ± 0.05^c^	110 ± 0.06^d^	7.06 ± 0.80^c^	190 ± 0.24^c^	0.34 ± 0.17^c^	72.6 ± 1.03^c^	9.34 ± 1.30^c^

**Table 3:** Impact of salicylic acid and tocopherol foliar spray on (no of leaves, leave length, leave fresh weight, leave dry weight, %moisture content, leave area ratio and leave area index) of *Triticum aestivum* under deep sowing stress

Varieties	Treatments	No. Of Leaves (NOL)	Leave Length (cm)	Leave Fresh Weight (mg)	Leave Dry Weight (mg)	%Moisture Content	Leave Area ratio (cm^2^)	Leaf area index (LAI)
**Pirsabak15**	Control	46.0 ± 0.00^a^	26.0 ± 0.44^a^	90.0 ± 0.01^b^	30.0 ± 0.06^b^	30.0 ± 4.77^b^	75.2 ± 0.37^bc^	0.90 ± 0.02^a^
	Deep Sowing	34.0 ± 2.82^c^	17.5 ± 0.40^b^	80.0 ± 0.06^c^	29.9 ± 0.04^b^	20.2 ± 5.55^c^	42.3 ± 0.60^cd^	0.42 ± 0.00^cd^
	Deep Sowing + Salicyclic acid	32.0 ± 2.82^c^	14.1 ± 0.83^c^	70.0 ± 0.12^d^	26.9 ± 1.23^c^	16.2 ± 5.10^d^	65.8 ± 0.47^c^	0.36 ± 0.02^d^
	Deep Sowing + alphaTocopherol	38.0 ± 2.82^c^	16.3 ± 1.54^c^	60.0 ± 0.04^e^	20.0 ± 1.98^d^	20.0 ± 3.36^c^	10.3 ± 0.09^d^	0.51 ± 0.05^c^
	DeepSowing + Salicyclicacid + Tocopherol	34.0 ± 2.82^c^	17.4 ± 0.51^a^	160 ± 0.44^a^	100 ± 0.95^a^	19.0 ± 5.64^c^	24.2 ± 0.55^d^	0.52 ± 0.05^c^
**Shankar**	Control	43.0 ± 2.82^b^	25.6 ± 0.44^a^	90.0 ± 0.21^b^	38.6 ± 2.01^b^	17.2 ± 6.09^c^	98.5 ± 0.76^a^	0.62 ± 0.03^b^
	Deep Sowing	38.0 ± 2.82^c^	16.9 ± 1.74^c^	50.0 ± 0.16^f^	20.9 ± 1.09^cd^	8.21 ± 3.98^d^	53.9 ± 0.53^bc^	0.37 ± 0.00^cd^
	Deep Sowing + Salicyclic acid	34.0 ± 2.82^c^	15.2 ± 1.57^c^	70.0 ± 0.00^d^	17.0 ± 1.36^d^	4.00 ± 3.09^e^	64.9 ± 1.32^c^	0.36 ± 0.05^d^
	Deep Sowing + alpha Tocopherol	42.0 ± 0.00^b^	18.1 ± 2.27^b^	90.0 ± 0.09^b^	30.9 ± 0.01^b^	28.2 ± 4.87^b^	75.5 ± 2.18^bc^	0.51 ± 0.08^b^
	DeepSowing + Salicyclicacid + Tocopherol	38.0 ± 2.82^c^	17.9 ± 2.12^b^	110 ± 0.48^ab^	20.6 ± 0.54^d^	68.8 ± 4.88^a^	81.5 ± 1.21^b^	0.49 ± 0.05^c^

### Impacts on antioxidant enzymes and physiological attributes

**Fig. 1:**
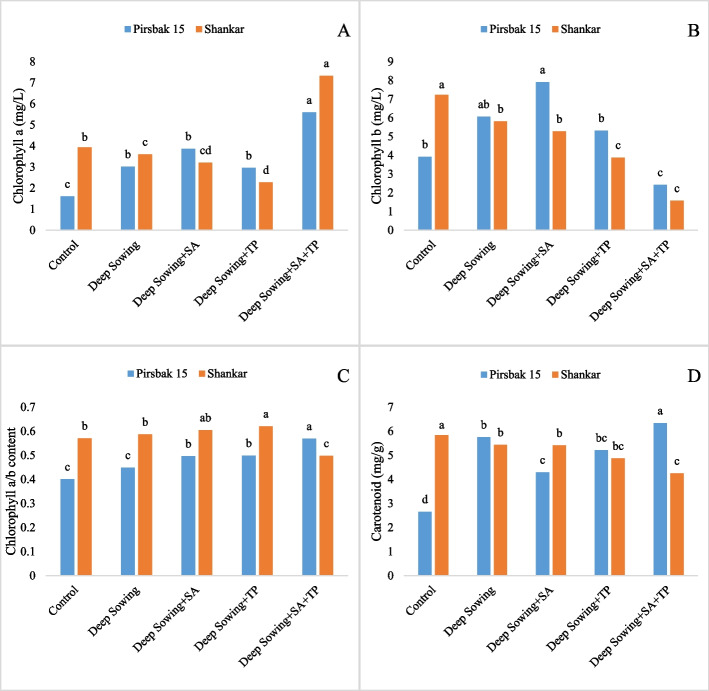
Effect of salicyclic acid and tocopherol foliar spray on chlorophyll a, b, a/b and carotenoid content under induced abiotic stress of deep sowing

**Fig. 2:**
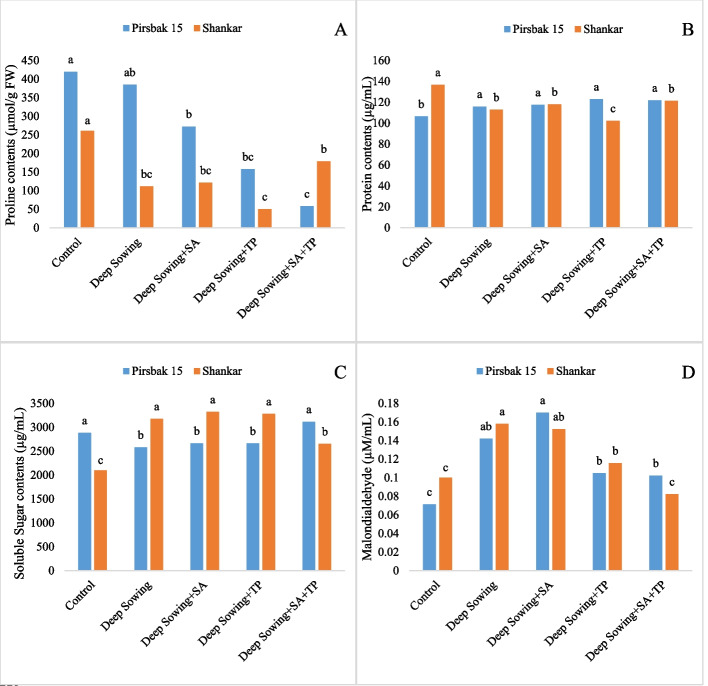
Effect of salicylic acid and tocopherol foliar spray on (**a**) Proline (**b**) Protein (**c**) Sugar and (**d**) MDA content under induced abiotic stress of deep sowing

**Fig. 3:**
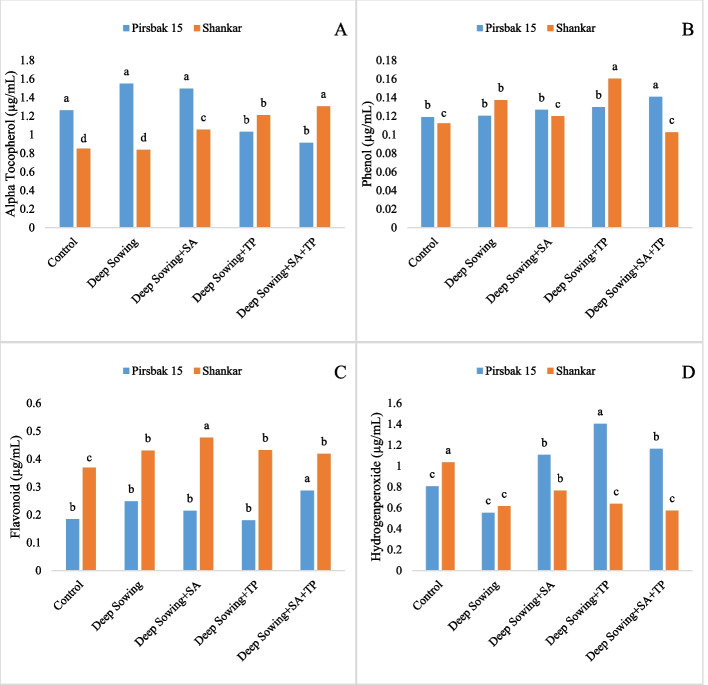
Effect of salicylic acid and tocopherol foliar spray on (**a**) alpha tocopherol (**b**) Phenol (**c**) Flavonoid and (**d**) hydrogen peroxide content under induced abiotic stress of deep sowing

**Fig. 4:**
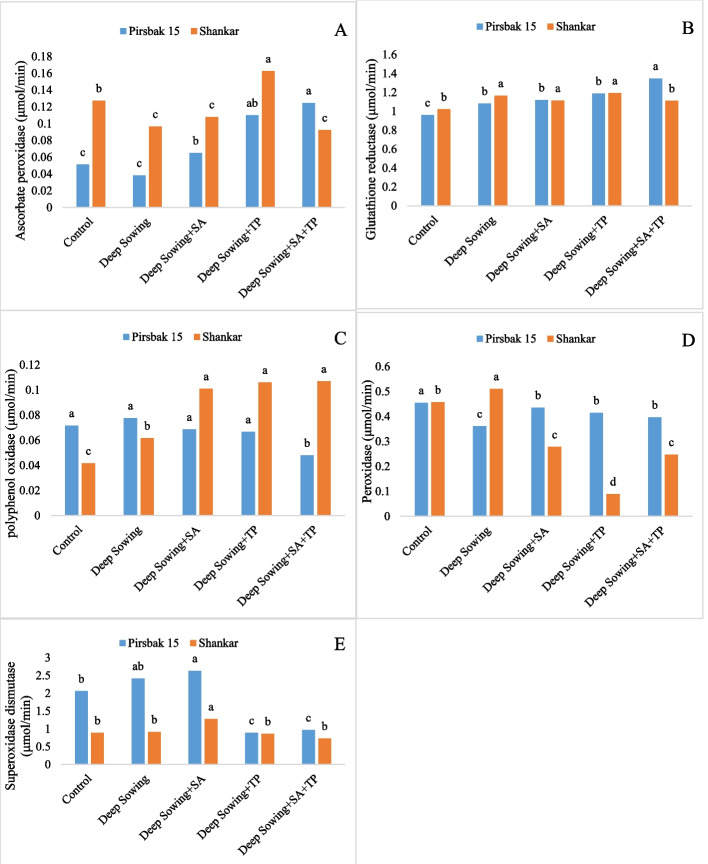
Effect of salicylic acid and tocopherol foliar spray on (**a**) APOX (**b**) GR (**c**) PPO (**D**) POD and (**e**) SOD content under induced abiotic stress of deep sowing

Stress caused by deep sowing led to a quick decrease in physiological characteristics and an increase in the activities of antioxidant enzymes. Compared to the control group, the levels of leaf photosynthetic pigments, such as chlorophyll a, chlorophyll b, and chlorophyll a/b contents, were reduced under the deep sowing conditions (Fig. 1). When subjected to deep sowing stress, the combined use of salicylic acid and tocopherol improved chlorophyll content. Plant photosynthetic pigments were the primary indicator of deep sowing stress due to their sensitivity and fragility. Studies revealed that salicylic acid exhibited displayed better results in boosting the levels of chlorophyll content as compared to α-tocopherol (Fig. 1). Deep sowing stress elevated the level of total soluble sugar; similarly, further increase was detected after foliar application of salicylic acid (Fig. 2). Besides acting as a source of nutrition it also plays a important role as osmo-tolerant by determining the structure of protein and stabilizing membrane structures. Under salicylic acid and alpha-tocopherol application a reduction was noted in proline concentration under deep sowing stress regimes. It was found that the protein content of the control declined noticeably (p<0.005) (Fig. 2). However, plants under stress accumulate proteins in a variety of ways. Fig. 3 showed that deep sowing stress caused a considerable decrease in H_2_O_2_ content. α-tocopherol and salicylic acid adjust stress by providing cellular protection. MDA and GR levels significantly improved (p<0.005) in all deep sowing stress-treated plants (Fig. 2, 4). Similar trend was also noted for GR, which was raised by the combined application of alpha tocopherol and salicylic acid. Antioxidant enzymes showed a marked increase by the foliar applications of salicylic acid and α-tocopherol. Both the wheat varieties grown in deep sowing indicated a significant enhancement in the activities of SOD, PPO and APX, whereas, POD activity showed a marked decline under deep sowing stress regimes (Fig. 4). Moreover, the concentration of PPO declined in all the groups under deep sowing stress regimes and improved with the foliar applications of salicylic acid and α-tocopherol (Fig. 4). Fig. 4 revealed a sharp decline in GR activity as the deep sowing stress increased. Under deep sowing stress conditions, exogenously applied salicylic acid and α-tocopherol improved physiological and agronomic attributes and activated the natural defense system of the plants (Fig. 4).

### Correlation, heatmap correlation and principal component analysis of different parameters measured for wheat germination

**Fig. 5:**
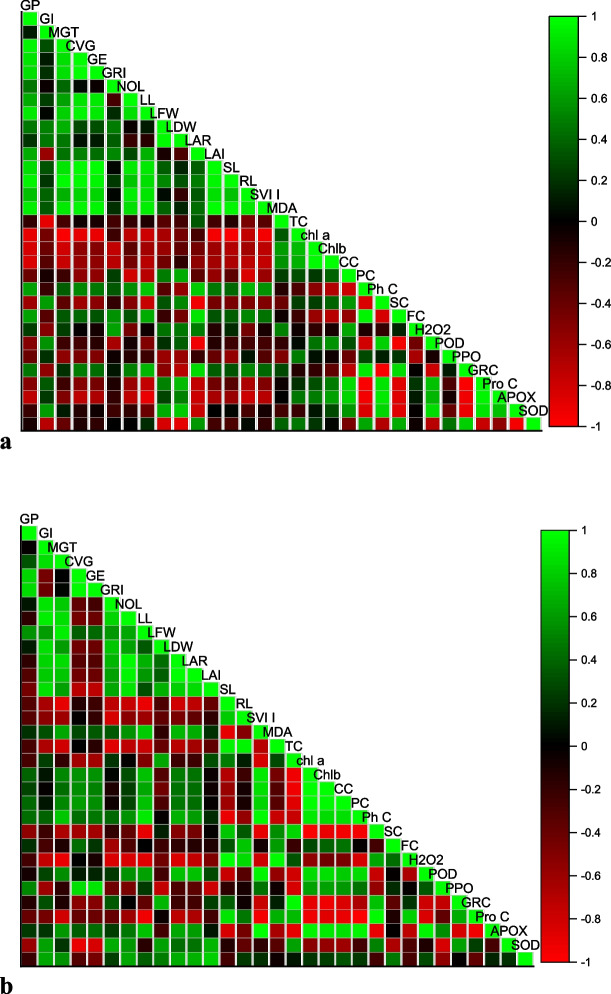
A Correlation between various germination attributes of Triticum aestivum L . var. Pirsabak 15 under deep sowing stress. Germination percentage (GP), Germination Index (GI), Mean Germination Time (MGT), Coffiecient Velocity of Germination (CVG), Germination Energy (GE), Germination rate Index (GRI), Number of Leave (NOL), Leave Length (LL), Leave Fresh Weight (LFW), Leaf Dry Weight (LDW), Leave Area Ratio (LAR), Leaf Area Index (LAI), Shoot Length (SL), Root Length (RL), Seed vigor Index I (SVI-I), Malondialdehde content (MDA), Tocopherol content (TC), Chlorophyll a content (Chl a) Chlorophyll b content (Chl b), Carotenoid Content (CC), Protein Content (PC), Phenolic Content (PhC), Sugar Content (SC), Flavnoid Content (FC), Hydrogen Peroxide (H 2 O 2 ), Peroxidase (POD), Polyphenol oxidase (PPO), Glutathione reductase (GRC), Phenolic content (PnC), Ascorbate peroxidase (APX), Superoxidase dismutase (SOD). B Correlation between various germination attributes of Triticum aestivum L. var. Shankar under deep sowing stress. Germination percentage (GP), Germination Index (GI), Mean Germination Time (MGT), Coffiecient Velocity of Germination (CVG), Germination Energy (GE), Germination rate Index (GRI), Number of Leave (NOL), Leave Length (LL), Leave Fresh Weight (LFW), Leaf Dry Weight (LDW), Leave Area Ratio (LAR), Leaf Area Index (LAI), Shoot Length (SL), Root Length (RL), Seed vigor Index I (SVI-I), Malondialdehde content (MDA), Tocopherol content (TC), Chlorophyll a content (Chl a) Chlorophyll b content (Chl b), Carotenoid Content (CC), Protein Content (PC), Phenolic Content (PhC), Sugar Content (SC), Flavnoid Content (FC), Hydrogen Peroxide (H 2 O 2 ), Peroxidase (POD), Polyphenol oxidase (PPO), Glutathione reductase (GRC), Phenolic content (PnC), Ascorbate peroxidase (APX), Superoxidase dismutase (SOD)

Alpha-tocopherol and salicylic acid were applied to deep-rooted wheat plants in order to quantify the relationship between the traits assessed on those plants (Fig. 5A and B). The association between 31 assessed qualities across the five treatments was presented using the correlation matrix. These coefficients computed a linear relationship and are outlier-sensitive. Germination parameters including GP, GRI, GI, MGT, GE, and CVG were positively correlated with growth traits like LDW, LFW, LL, SL, RL, LAI, and LAR, whereas biochemical parameters like H_2_O_2_, SOD, POD, APX, Chl a, Chl b, CC, and MDA were negatively correlated with growth traits. Due to the scavenging nature and formation of reactive oxygen species in cells, various metrics as well as enzymatic activities in particular elevated under deep sowing stress conditions.

**Fig. 6:**
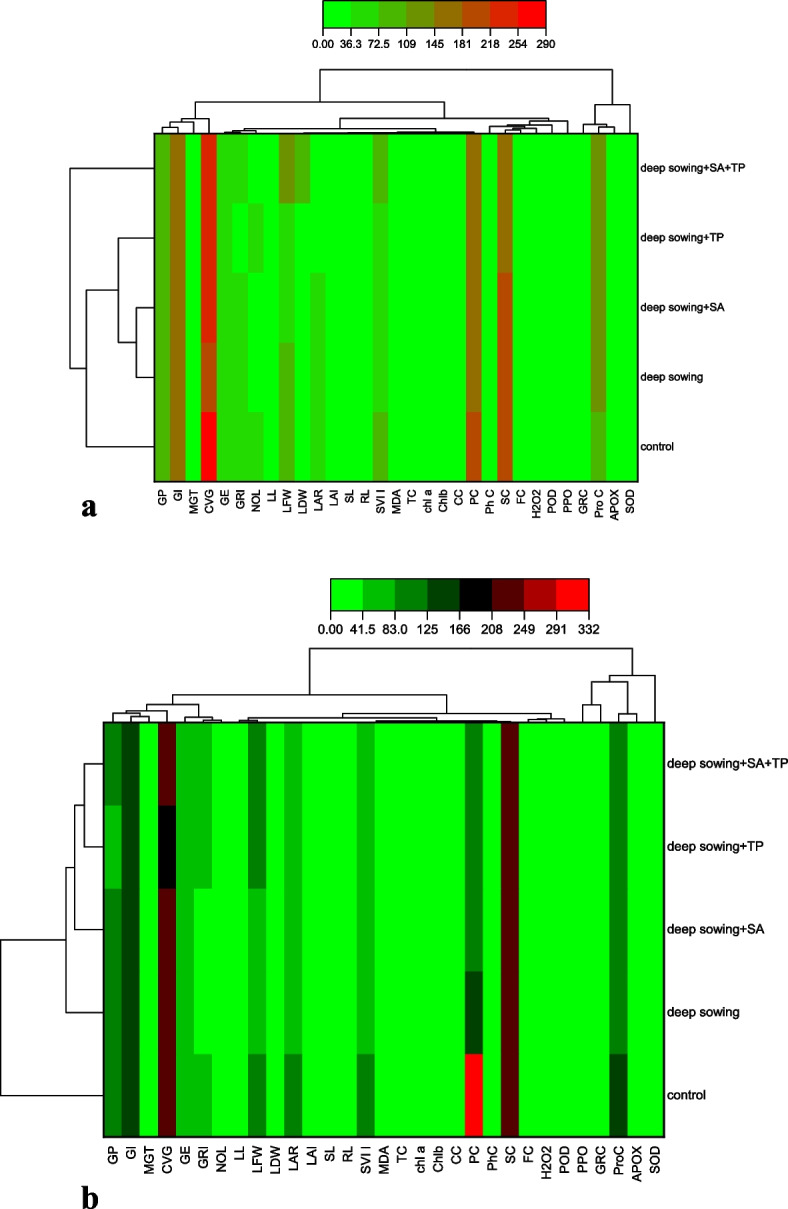
A Heatmap histogram correlation between various germination attributes of Triticum aestivum L. var. Pirsabak 15 under deep sowing stress. Germination percentage (GP), Germination Index (GI), Mean Germination Time (MGT), Coffiecient Velocity of Germination (CVG), Germination Energy (GE), Germination rate Index (GRI), Number of Leave (NOL), Leave Length (LL), Leave Fresh Weight (LFW), Leaf Dry Weight (LDW), Leave Area Ratio (LAR), Leaf Area Index (LAI), Shoot Length (SL), Root Length (RL), Seed vigor Index I (SVI-I), Malondialdehde content (MDA), Tocopherol content (TC), Chlorophyll a content (Chl a) Chlorophyll b content (Chl b), Carotenoid Content (CC), Protein Content (PC), Phenolic Content (PhC), Sugar Content (SC), Flavnoid Content (FC), Hydrogen Peroxide (H 2 O 2 ), Peroxidase (POD), Polyphenol oxidase (PPO), Glutathione reductase (GRC), Phenolic content (PnC), Ascorbate peroxidase (APX), Superoxidase dismutase (SOD). B Heatmap histogram correlation between various germination attributes of Triticum aestivum L. var. Shankar under deep sowing stress. Germination percentage (GP), Germination Index (GI), Mean Germination Time (MGT), Coffiecient Velocity of Germination (CVG), Germination Energy (GE), Germination rate Index (GRI), Number of Leave (NOL), Leave Length (LL), Leave Fresh Weight (LFW), Leaf Dry Weight (LDW), Leave Area Ratio (LAR), Leaf Area Index (LAI), Shoot Length (SL), Root Length (RL), Seed vigor Index I (SVI-I), Malondialdehde content (MDA), Tocopherol content (TC), Chlorophyll a content (Chl a) Chlorophyll b content (Chl b), Carotenoid Content (CC), Protein Content (PC), Phenolic Content (PhC), Sugar Content (SC), Flavnoid Content (FC), Hydrogen Peroxide (H 2 O 2 ), Peroxidase (POD), Polyphenol oxidase (PPO), Glutathione reductase (GRC), Phenolic content (PnC), Ascorbate peroxidase (APX), Superoxidase dismutase (SOD)

A heat-map histogram between the variables of the various treatments examined in this study was also displayed (Fig. 6A and B). These variables were producing results that line up with what the correlation analysis showed. Between treatments, two separate clusters were produced. The first cluster included the controlled group, deep sowing, deep sowing + SA, deep sowing + TP, and deep sowing + SA + TP for the var. Pirsabak 15, whereas the second cluster was formed for the var. Shankar (Fig. 6A and B).

**Fig. 7:**
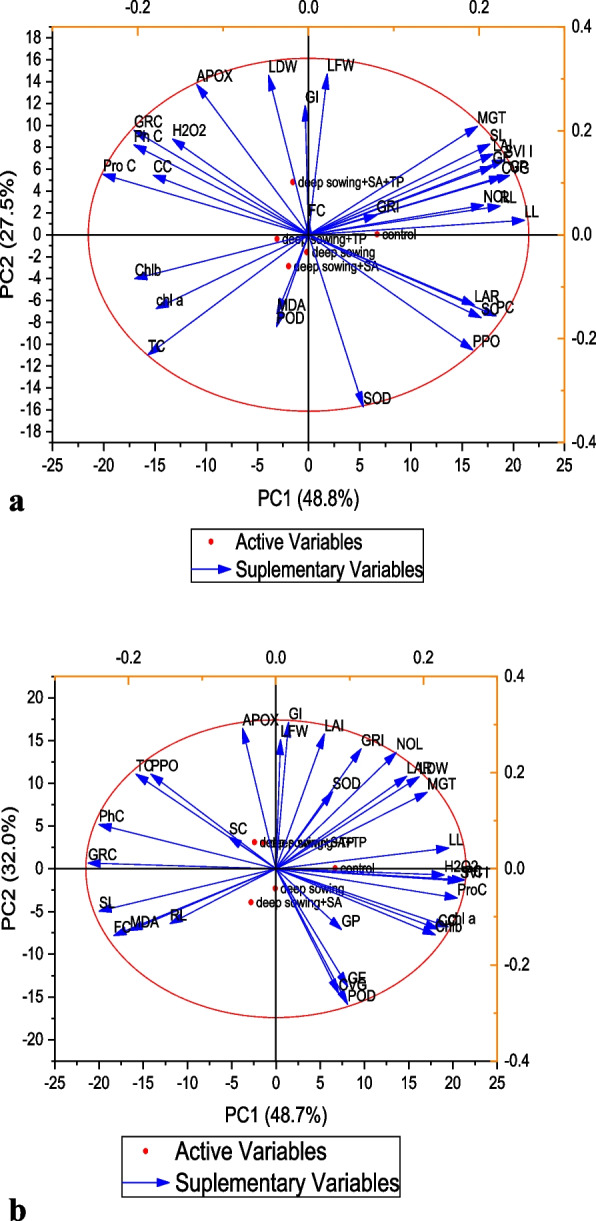
A Loading Plot of Principal component analysis (PCA) on various germination attributes of Triticum aestivum L. var. Pirsabak 15 under deep sowing stress. Germination percentage (GP), Germination Index (GI), Mean Germination Time (MGT), Coffiecient Velocity of Germination (CVG), Germination Energy (GE), Germination rate Index (GRI), Number of Leave (NOL), Leave Length (LL), Leave Fresh Weight (LFW), Leaf Dry Weight (LDW), Leave Area Ratio (LAR), Leaf Area Index (LAI), Shoot Length (SL), Root Length (RL), Seed vigor Index I (SVI-I), Malondialdehde content (MDA), Tocopherol content (TC), Chlorophyll a content (Chl a) Chlorophyll b content (Chl b), Carotenoid Content (CC), Protein Content (PC), Phenolic Content (PhC), Sugar Content (SC), Flavnoid Content (FC), Hydrogen Peroxide (H 2 O 2 ), Peroxidase (POD), Polyphenol oxidase (PPO), Glutathione reductase (GRC), Phenolic content (PnC), Ascorbate peroxidase (APX), Superoxidase dismutase (SOD). B Loading Plot of Principal component analysis (PCA) on various germination attributes of Triticum aestivum L. var. Shankar deep sowing stress. Germination percentage (GP), Germination Index (GI), Mean Germination Time (MGT), Coffiecient Velocity of Germination (CVG), Germination Energy (GE), Germination rate Index (GRI), Number of Leave (NOL), Leave Length (LL), Leave Fresh Weight (LFW), Leaf Dry Weight (LDW), Leave Area Ratio (LAR), Leaf Area Index (LAI), Shoot Length (SL), Root Length (RL), Seed vigor Index I (SVI-I), Malondialdehde content (MDA), Tocopherol content (TC), Chlorophyll a content (Chl a) Chlorophyll b content (Chl b), Carotenoid Content (CC), Protein Content (PC), Phenolic Content (PhC), Sugar Content (SC), Flavnoid Content (FC), Hydrogen Peroxide (H 2 O 2 ), Peroxidase (POD), Polyphenol oxidase (PPO), Glutathione reductase (GRC), Phenolic content (PnC), Ascorbate peroxidase (APX), Superoxidase dismutase (SOD)

The PCA (principal component analysis) was utilized to connect the morpho-physiological traits and antioxidant enzymes under induced deep planting stress. The experimental datasets underwent PCA, which contained 22 morphological and 17 physio-biochemical variables in addition to the control and treatment groups (Fig. 7A and B). The results demonstrated that the respected treatments were all evenly dispersed over the whole datasets. Whether or not salicylic acid and alpha-tocopherol were given topically, the distribution of all the dataset’s elements clearly showed that deep sowing had a significant influence on a number of morph-physiological and biochemical properties. All variables were scattered according to the PCA plot, which showed that deep planting significantly affected these characteristics. The results showed that the first two major factors accounted for 57% of the data set’s overall volatility.

## Discussion

Germination percentage were also influenced by changes in sowing depth, Likewise the other parameters it showed minimum counts under deep sowing at 6 cm and were highest at 2 cm depth. Our findings were consistent with the results of [16] on chick pea. The harmful effects of deep sowing depth were described by [17] who discovered that the sprouting of seedlings reduced as the sowing depth increased in cotton plants. Seeds sown deeper in the soil require more power to push their shoots above the soil surface. According to the proposal, shallow sowing depths should be adopted with similar seeds. Supporting evidences were also established by [18] in *Cinnamomum tamala*. Decline in plant agronomic features have been mainly caused by stomatal closure during osmotic stress regimes [19]. Oxidative stress induces root elongation in stress tolerant species. Occasionally, moderate osmotic stress has no obvious harmful impact on root growth [20]. With foliar applications, the photosynthetic pigments of plant are adjusted by reducing the rate at which hydrogen peroxide is produced and enhancing the level of phenolic compounds, thus enabling the stressed plants to keep their physiological characteristics stable [21]. Deep sowing significantly reduced the carotenoid contents at p level *<* 0.005. On exposure to stress conditions accumulation of organic solutes in the cytoplasm takes place; including glycine betaine, proteins and proline that support stressed plants in the osmotic adjustment of organic solutes [22].In addition, a similar spike was noticed in proline content under deep sowing stress, while the lowest was concentration was found in the control group. Likewise, Proline accumulation under exposure to deep sowing stress has been observed by a number of researchers [10, 22, 23].There was a relatively high level of protein content in all treatments that received foliar applications of salicylic acid and tocopherol. The responses of plants to deep sowing stress are clearly influenced by protein [24].The upsurge in MDA contents under deep sowing stress in wheat was parallel to that observed in ornamental grass by [25] and rice [26].The improvements in the activities of antioxidants enzymes could be due to deep sowing stress and the combined regulatory effect of salicylic acid and α-tocopherol. To combat the harmful effect of abiotic stress, plants need antioxidant enzymes [27].Together with activating antioxidant enzymatic systems, oxidative stress prompts the stressed plants to accumulate soluble sugars, proline and soluble proteins in the cytoplasm to maintain osmoregulation [28, 29].

## Conclusions

The studies conducted on seed germination have shown that deep sowing negatively affects germination and emergence. Maximum seed germination and seed vigor index were observed in the seeds sowed at a depth of 2 cm. On the other hand, the seeds sowed at a depth of 6 cm showed poor agronomic and physiological traits of the emerging wheat seedlings. However, the application of salicylic acid and tocopherol led to significant improvement in the number of leaves, leaf area ratio, and shoot height. Moreover, salicylic acid and tocopherol trigger various antioxidative defence mechanisms which significantly reduce the oxidative damage caused by ROS, both enzymatically and non-enzymatically. Their role in making several varieties of wheat stress-tolerant has been identified through the activation of antioxidant enzymes and accumulation of osmoprotectants. In a comparative study, salicylic acid was found to be more effective than tocopherol in reducing oxidative stress in wheat varieties.

## Methods

### Site description and experimental layout

Pot experiment was set in the net house of the department of Botany, University of Peshawar, Province Khyber Pakhtunkhwa, Pakistan; in spring season during the year 2021. Randomized block design layout was followed with three pot duplicates. The experimental site is located at an altitude of 450 m, the area has sub-humid environment with severe weather (mild winter: 18.35 °C; hot summer: 40.8 °C). Wheat varieties Pirsabak 15 and Shankar were obtained from the NIFA (Nuclear Institute of Food and Agriculture). After being surface-sterilized with 95% ethanol, seeds were planted in clay pots with a diameter of 20.0 cm in length, 2.0 cm in thickness, and 18.0 cm upper-lower diameter. The pots were filled with 3 kg of soil and sand with 2:1. The 1st is controlled group; while in the others replicates 6 cm deep sowing in the soil mixture. After seedling emergence, 150 mg/L of exogenous growth mediators (Salicylic Acid and Tocopherol) were applied to the plants. Agronomic aspects of vegetative development, including data on germination, were noted. To evaluate physio-biochemical and enzymatic properties, the residual plants were stored at 4 °C in the freezer.

### Agronomic and germination characteristics

Agronomic and germination indices including germination rate index (GRI), mean germination time (MGT), germination energy (GE), coefficient velocity of germination (CVG), germination index (GI), leaf area ratio (LAR), and leaf area index, were examined using the techniques of [30]. While leaf area index (LAI), seed vigor indices (SVIs), germination percentage (GP), and % moisture content were calculated via following [31].

1 $$\text{MGT}=\frac{\sum \text{fx}}{\sum \textrm{f}}$$

The number of seeds that germinated on day x is denoted by the letter “f.“

2 $$\text{GRI}=\frac{\text{G}1}1+\frac{\text{G}2}2+\cdots\cdots+\frac{\text{Gx}}{\textrm{x}}$$

The proportion of seeds that germinated on the first day day after sowing is denoted by G1, whereas the percentage on the second day after sowing is denoted by G2.

3 $$\text{GI} = (10 \times \text{n}1) + (9 \times \text{n}2) + ----- + (1 \times \text{n}10)$$

In the above Eqs. 10, 9,-------1 represented the number of days of germination, respectively, whereas n1, n2,…, and n10 represented the No. of seeds that germinated on each day.

4 $$\text{CVG}=\text{N}1\pm\text{N}2\pm\dots.\pm\frac{\text{NX*N}1\text{T}1+\dots .\text{NxT}}{100}$$

Where “N” represents the daily seed germination rate, “T” represents the number of days from planting, and “N” represents the daily seed germination rate.

5 $$\text{GE}=\frac{\text{X}1}{\textrm{X}2}+\frac{(\text{X}2-\text{X}1)}{\textrm{Y}2}\dots\dots .+\frac{(\text{Xn}-\text{Xn}-1)}{\textrm{Yn}}$$

The No. of days between sowing and the last (nth) counting date is Yn, and the final germination on that day is denoted by the number Xn.

6 $$\text{SVI}-\text{I}=\text{seedling length}\left(\text{cm}\right)\text{*seedling germination }{\%}\text{age}$$

7 $$\text{SVI}-\text{II}=\text{Seedling dry weight} \left(\text{mg}\right) \text{x Seed germination }{\%}\text{age}$$

8 $$\text{LAI}=\frac{\text{Leaf area} }{\textrm{Land area} }$$

9 $$\text{Leaf area ratio}=\frac{\text{leaf area}}{\textrm{final plant dry weight}}$$

10 $$\text{Root}-\text{shoot ratio}=\frac{\text{Root dry mass}}{\textrm{shoot dry mass}}$$

11 $${\%}\text{moisture content}=\frac{\text{Wet weight of sample}-\text{dry weight of sample}}{\textrm{Dry weight of sample}}$$

### Physiological and biochemical attributes

#### Leaf Photosynthetic Pigment

The methodology of [32] was followed to assess photosynthetic pigments including Chlorophyll b and Chlorophyll a. Carotenoid (CAR) contents were quantified by using the protocol of [33] by applying the following equations:

12 $$\text{Chl a}=\left\{12.7\left(\text{OD}663\right)-2.69 \left(\text{OD}645\right)\right\}\times \text{V}\div 1000\times \text{W}$$

13 $$\text{Chl b}=\left\{22.9\left(\text{OD}645\right)-4.68 \left(\text{OD}663\right)\right\}\times \text{V}\div 1000\times \text{W}$$

14 $$\text{Carotenoid}=\text{DA}480+(0.114\times \text{DA}663)-(0.638\times \text{DA}645)$$

Where V is the extract level (in millilitres), W is the weight of the fresh leaves, and DA is the optical density at the specified wavelength.

### Total proline content (TPC) and soluble protein content (SPC)

TPC of leaves was quantified using the method described by [34]. Meanwhile, the protocol of [35] was pursued to quantify the amount of soluble proteins. Both contents’ values were calculated using equation [14].

15 $$\text{Protein}{\%}(\text{W}/\text{W})=\text{Cp}\times \text{V}\times \text{DF}\div \text{Wt}$$

Where wt is the weight of the leaves, DF is dilution factor, V is the volume of the buffer lysis, and Cp is the protein concentration (mg L1) (mg).

### Soluble Sugar Content (SSC) and hydrogen peroxide content (H_2_O_2_)

The technique employed by [36] was followed to determine soluble sugar content of the leaves. The methodology of [37] was applied to assess H_2_O_2_ activity in a method similar to that. The OD of sugar and H_2_O_2_ were measured at wavelengths of 420 and 390 nm, respectively.

### Phenolic content (PC) and flavonoid content (FC)

The PC of the leaves was measured using the technique proposed by [38]. Flavonoid content was quantified by following the methodology of [39]. The optical densities (ODs) of phenolic and flavonoid content were determined at 730 nm and 430 nm, respectively.

### Malondialdehyde (MDA) and alpha tocopherol content assay

The methodology proposed by [40] was used to carry out the MDA content. At 530 nm, the OD was measured. To quantify alpha-tocopherol content in leaf the methodology [41] was followed. MDA content were estimated using the following formulas:

16 $$\text{MDA}\left(\text{nmol}\right)=\text{D}(\text{A}532\text{nm}-\text{A}600\text{nm})/1.56\times 105$$

### Antioxidant enzymatic assays

The conventional method of [42] was used to measure SOD (superoxide dismutase) activity using a spectrophotometer at 560 nm. The approach of [33] was utilized to evaluate glutathione reductase (GR) and peroxidase (POD) activity at 420 and 340 nm, respectively. Ascorbate peroxidase (APX) activity was carryout via using the methodology of [43].

17 $$\text{EA}=\varDelta \text{A}\times \text{Total Volume}\div \varDelta \text{t}\times \in \times \text{i}\times \text{enzyme sample volume}$$

Where ∆t is the incubation duration, E is the substrate’s absorbance coefficient and ∆A is the change in absorbance.

### Statistical analysis

In order to calculate mean value and standard error from the gathered data, Microsoft Excel 2010, US, was utilized. Co-Stat Window version 6.3 was used to conduct an analysis of variance (ANOVA) to discover significant variations between treatments. Standard methods were used to compute the mean and standard error, and a LSD (least significant difference) test was applied at the 0.05 significance level and the results were displayed in letters (AE). R Studio 8.1 was used to carry out the correlation study.

## Data Availability

All data generated or analysed during this study are included in this published article.

## Abbreviations

GI: Germination Index
GE: Germination Energy
GP: Germination percentage
GR: Germination rate
GRI: Germination rate index
MDA: Malondialdehyde
MPa: Mega Pascal
MGT: Mean germination time
PEG: Polyethylene glycol
RH: Relative humidity
SVI: Seed vigor index
UV: Ultra Violet
OD: Optical density
NM: Nanometer
POD: Peroxidase
GR: Gluthinone reductace
APX: Ascorbate peroxidase
SOD: Superoxide dismutase
SA: Salicylic acid
